# Synergy of arsenic with smoking in causing cardiovascular disease mortality: A cohort study with 27 follow-up years in China

**DOI:** 10.3389/fpubh.2022.1012267

**Published:** 2022-12-14

**Authors:** Xin-Hua Jia, Zheng Su, Fang-Hui Zhao, Qing-Hua Zhou, Ya-Guang Fan, You-Lin Qiao

**Affiliations:** ^1^The State Key Laboratory of Molecular Vaccinology and Molecular Diagnostics, National Institute of Diagnostics and Vaccine Development in Infectious Diseases, School of Public Health, Xiamen University, Xiamen, Fujian, China; ^2^Department of Epidemiology, National Cancer Center/National Clinical Research Center for Cancer/Cancer Hospital, Chinese Academy of Medical Sciences and Peking Union Medical College, Beijing, China; ^3^Department of Tobacco Control and Prevention of Respiratory Disease, Center of Respiratory Medicine, China-Japan Friendship Hospital, Beijing, China; ^4^WHO Collaborating Center for Tobacco Cessation and Respiratory Diseases Prevention, Beijing, China; ^5^National Clinical Research Center for Respiratory Diseases, Beijing, China; ^6^Institute of Respiratory Medicine, Chinese Academy of Medical Sciences, Beijing, China; ^7^National Center for Respiratory Medicine, Beijing, China; ^8^Sichuan Lung Cancer Center, Sichuan Lung Cancer Institute, West China Hospital, Sichuan University, Chengdu, China; ^9^Tianjin Key Laboratory of Lung Cancer Metastasis and Tumor Microenvironment, Tianjin Lung Cancer Institute, Tianjin Medical University General Hospital, Tianjin, China; ^10^Center for Global Health, School of Population Medicine and Public Health, Chinese Academy of Medical Sciences and Peking Union Medical College, Beijing, China

**Keywords:** inhaled arsenic, cigarette smoking, cardiovascular disease mortality, cohort, synergy effect

## Abstract

**Background:**

To explore the patterns of the exposure-response relationship between arsenic exposure and cardiovascular disease (CVD) mortality and investigate the effect of cigarette smoking on the association.

**Methods:**

Seven thousand seven hundred thirty-five tin miners with at least 10 years of arsenic exposure were enrolled since 1992 and followed up for 27 years. Each individual's air arsenic exposure at workplace was calculated by time weighted average arsenic concentration × exposure months. Detailed information on smoking was collected at baseline, and information on smoking status was collected for five consecutive years from 1992 to 1996. Hazard ratio (HR) and 95% confidence interval (CI) for the risk of CVD were estimated using Cox proportional hazards models.

**Results:**

A total of 1,046 CVD deaths occurred in this cohort over 142,287.7 person-years of follow up. We firstly reported that for equal cumulative exposure, participants exposed to higher concentrations over shorter duration had a higher risk of CVD mortality than those exposed to lower concentration over longer duration. The HR and 95% CI were 1.38 (95%CI: 1.03–1.85) in participants exposed to arsenic concentration (45.5–99.5 mg/m^3^), 1.29 (95%CI: 1.02–1.67) in 99.5–361.0 mg/m^3^. Further, participants with age at first exposure <18 years had a significantly higher risk of morality from CVD, cerebrovascular and heart diseases than those with ≥18 years. Finally, all synergy indices were greater than 1 (range, 1.11–2.39), indicating that the joint effect of arsenic exposure and cigarette smoking on CVD mortality was greater than the sum of their individual effect.

**Conclusions:**

Exposure to air arsenic at workplace is adversely associated with mortality from CVD, especially among smokers younger than 18 years and smokers.

## Background

Environmental exposure to high concentration arsenic had adverse health effects including cancers (1), cardiovascular diseases (CVD) (2), neurotoxicity, diabetes mellitus, and other diseases (3, 4). The relationship between low concentration arsenic and the risk of deaths from any CVD is of great public health significance. However, no studies have reported a pattern of exposure-response relationships for arsenic-related CVD that is conceptually similar to the “dose-rate effectiveness factor.” It describes the effect on disease risk of the total radiation dose delivered at lower (persistent or highly fractionated) dose rates compared to the effect at higher (or acute) dose rates for a given type of radiation (5).

In arsenic-related CVD studies, smoking information should be taken into consideration. This is because cigarette smoking is also a risk factor for CVD and accounts for 10% of CVD-related deaths worldwide (6). Although the effect of smoking cessation on CVD risk reduction has been well documented (7), However, there is a lack of smoking information in most studies of arsenic-related CVD. Only evidence from a US population-based study (8) and the Bangladesh prospective cohort (9) support that there is a synergistic relationship between cigarette smoking and arsenic exposure on mortality from CVD.

In 1973, the nationwide survey on the cause-of-death in China revealed that Gejiu, a city located in Yunnan Province in southern China, had the highest male lung cancer mortality rate in all of China. The disease burden was involved principally in the tin miners around the city of Gejiu. Since 1986 the Nation Cancer Institute/National Institute of Health had collaborated with scientists at Cancer Hospital, Chinese Academy of Medical Sciences and the Labor Protection Institute to conduct a number of case-control and cohort studies among the tin miners, they found that the phenomenon that these miners lacked effective protective equipment and the factory lacked necessary ventilating facilities, which made miners exposed to extremely high concentrations of environmental radon and arsenic pollutants (accompanied by the mining process of tin ore) (10–13). In 1992, scientists built a prospective and occupational cohort to explore the hazard effect of environmental pollutants (airborne radon and arsenic) on human health (cancer and CVD) (14). In previous studies, we have established that the relationship between environmental pollutants and lung cancer (15–17). However, few studies tried to report the association between pollutants and CVD among tin miners.

With an extend follow-up, we firstly explore the patterns of the exposure-response relationship between arsenic exposure and CVD mortality in this prospective cohort study; And, with information on smoking at multiple points in time, we also investigated the synergy between arsenic exposure and cigarette smoking on CVD mortality.

## Methods

### Study design and participants

**Figure 1:**
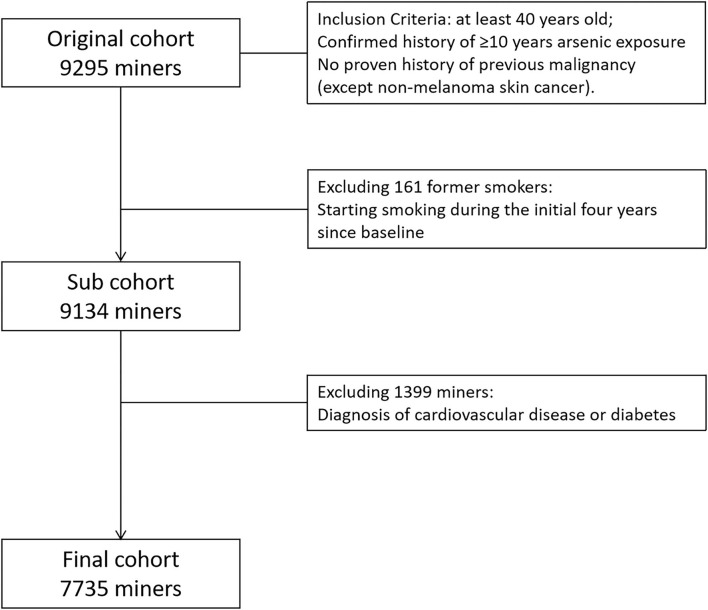
Flow chart of selections of the participants included in final analysis.

This cohort is a combined cohort. Briefly, a total of 3,278 tin miners with at least 10 years of arsenic exposure were enrolled into the arsenic occupational cohort, and 6,017 tin miners with at least 10 years of underground radon exposure were included into the radon occupational cohort. Given tin miners exposed to both radon and arsenic simultaneously, we also collected air arsenic exposure data in the radon cohort. Therefore, we combined arsenic cohort and radon cohort together into the full cohort (9,295 tin miners). Then, 161 former smokers were excluded, due to instability of smoking status. One thousand three hundred ninety-nine tin miners with a diagnosis of CVD or diabetes at baseline were also excluded. Finally, 7,735 tin miners were included into the final analysis (Figure 1).

### Arsenic exposure

A detailed occupational history included information on job title, worksite, and starting/stopping dates by year for eachjob held at the Yunnan Tin Corporation (YTC) for at least 1 year.

Since 1986, we have conducted lots of researches about the arsenic-related health effects in these tin miners. To measure the cumulative arsenic exposure of individual miners, we collected arsenic exposure concentration in the working environment of different workplaces in different years, then investigated the occupational history of miners including the workplaces and working hours. Finally, we multiply the above two factors to obtain individual arsenic exposure data. Therefore, the descriptions of arsenic exposure in all our studies are the same and detailed information had been reported previously (11). Briefly, exposure to arsenic containing ore dust (arsenic exposure) was quantitatively estimated from industrial hygiene data obtained separately for each of the four mines (Laochong, Makuang, Songkuang, and Kafang) over five eras (before 1952, 1952–1959, 1960–1969, 1970–1979, and after 1980) and three smelters in three periods (1959–1980, 1967–1970, and 1969–1975).

Airborne dust concentrations at the YTC were first measured in the 1950s, when dry drilling was common and large scale mining was taking place. Underground airborne dust concentrations reached 20–102.6 mg/m^3^, then fell appreciably to about 6.2 mg/m^3^ around 1964 when the practice of wet drilling became widespread. Typically, arsenic represents about 1.34% of the mined ore by weight as trivalent arsenic (As_2_O_3_).

Airborne arsenic concentration before 1970 was estimated as the product of arsenic content of dust times the concentration of dust in the air. Airborne time weighted average arsenic concentration was calculated for ambient air in the mining environment for each mine and smelter based on the arsenic content of dust, the concentration of dust in the air, and the specific time. After 1970, direct air arsenic concentrations were measured. Calculated or measured mean values by era are shown in Table 1.

**Table 1:** Calculated or measured mean values of Arsenic Concentration by era.

**Era**	**Arsenic (mg/m^3^)**	**Mines/smelters**	**Number of miners**
< 1951	0.42	Laochang	170
1952–1959	0.06	Makuang	217
1960–1969	0.04	Songkuang	388
1970–1979	0.03	Kafang	172
>1980	0.01	The first smeltery	874
		The second smeltery	257
		The third smeltery	590

Individual worker exposure to arsenic for each job was estimated by using an index (index of arsenic exposure month or IAEM) as follows: IAEM = time weighted average arsenic concentration (mg/m^3^) × exposure months. One IAEM is equivalent to exposure for 25 days (one month) at 1 mg/m^3^. If total inspired air per day is estimated at 3.6 m^3^ and 100% absorption is assumed then one IAEM is equivalent to 90 mg total arsenic exposure.

### Radon exposure

Similarly, radon exposure data was also obtained by multiplying the radon concentration in the workplace by the working time of individuals in the environment. Our previous researches about the relationship between radon and lung cancer had described radon exposure in detail (14). Briefly, exposure to radon daughters was estimated from industrial hygiene values measured or estimated for each individual mine (Laochang, Makuang, Songkuang, and Kafang) and era (<1952, 1953–1972, and >1973).

Radon daughter measurements were first made at the four principal mining areas of YTC in 1972 when a radon problem was initially recognized.

Estimates were made for exposures that occurred prior to the actual collection of industrial hygiene data. Large-scale tunnel production started at the YTC in 1953. In order to estimate exposure values for the era prior to 1953, 117 samples were taken from 13 small pits that operated pre-1949 and were still available for testing.

With the assistance of old miners, primitive mining work environments were simulated in these pits for the testing. Scientists from the Labor Protection Institute of the YTC and the Institute of Radiomedicine of the Chinese Academy of Medical Sciences used values from the 413 samples for radon daughters measured in the mines in 1972 as the basis for estimating values for the era 1953–1972.

Since then, systematic monitoring of radioactivity in the YTC has been carried out, and over 26,000 samples have been collected and analyzed.

Median values (and ranges) for radon daughters as WL (working level) for the three eras are summarized in Table 2.

**Table 2:** Calculated or measured mean values of Radon Concentration by era.

**Era**	**Radon daughter [WL]**	**Mines**	**Number of miners**
< 1952	1.64 (0.65–2.65)	Laochang	1,305
1953–1972	1.94 (0.23–4.27)	Makuang	1,069
1973–1980	0.77 (0.52–1.14)	Songkuang	1,579
		Kafang	1,114

Mine-, job-, and era-specific exposure to radon daughters was estimated as WLM for each job for each subject using the following formula:


$$\begin{array}{l} WLM\;=(285\;days/year\;\times \;8\;h/day)/\;170\;h/month\;\times WL\\ \;\times exposure\;time\;(in\;years). \end{array}$$

Cumulative exposure was estimated by summing the exposure levels from each job before diagnosis for cases or to the matched case for controls.

Definition of cessation of arsenic exposure: For retired miners, the date of cessation of exposure was the date of retirement; for unretired workers, the date of cessation was set to 1996. This date was selected because decades of engineering protection measures dramatically reduced arsenic exposure levels similar to the Chinese national hygiene standard levels by 1996 (18).

Effect modification factors: We defined time since last exposure (TSE), attained age (AA), and age at first exposure (AFE) as effect modification factors of arsenic-related CVD.

### Tobacco use

All miners were enrolled at baseline (in 1992) and a self-designed and standardized questionnaire was administrated to each participant to collect information on smoking through a face-to-face interview with well-trained local health workers. A total of 13 questions included information on smoking status, what types of tobacco products they used (cigarettes, waterpipe, and long-stem pipe), the age they started/stopped smoking, number of cigarettes/waterpipe/long-stem pipe smoked per day. According to the smoking status at baseline, we divided miners into smokers, former smokers, and never smokers. At baseline, individuals who had smoked regularly for 6 months or longer at any time in their lives were classified as smokers, and those who have a smoking duration of less than 6 months were considered never smokers and smokers who ceased smoking at enrollment were former smokers.

In addition, to test the stability of smoking status, a self-designed questionnaire was administrated to each miner to collect information on smoking status and type of tobacco for five consecutive years from 1992 to 1996. The change of smoking status for at least two consecutive years was identified as “the real behavior change” from 1992 to 1996.

Then, results showed that only 110 of 6,899 (1.6%) of current smokers quit, and only 31 of 1,463 (2.1%) of never smokers started smoking. However, 161 of 933 (17.3%) former smokers returned to smoking.

The impact of type of cigarette was not considered in final analysis. In addition, we calculated a cigarette-equivalent variable that combined cigarette and water-pipe use by adjusting moderately that 1 g water-pipe = 1 g cigarette (12, 14, 19).

### Outcome information

The primary outcome was deaths from CVD, defined as ICD-10 (international classification of diseases, 10th revision codes) I05-I69 in cohort participants from baseline to December 31, 2018. The secondary outcome was deaths from cerebrovascular diseases (ICD-10: I60–I69) and heart diseases (ICD-10: I05–I09, I20–I28 and I30–I52). Other causes of death were viewed as a competing event.

Causes of deaths were identified from the local cancer registration agency, medical record system, death cause systems of public security bureau, and funeral parlor, and face-to-face interviews with relatives and workmates of the participants. In the process of information extracting, participants' name, age, work units and home address were taken into consideration. By the end of December 31, 2018, 136 participants (1.8%) were lost to follow-up, with a follow-up rate of 98.2%. Total causes of death and data sources in this cohort was seen in Supplementary Table S1.

### Statistical analysis

We tabulated baseline frequencies and percentages by demographic factors for participants in the different arsenic concentration groups, which all miners were equally classified as four subgroups by arsenic concentration: 1,903 participants in <2.5 mg/m^3^ (Group 1), 1,876 in 2.5–4.4 mg/m^3^ (Group 2), 2,032 in 4.5–13.9 mg/m^3^ (Group 3), 1,924 in ≥14.0 mg/m^3^ (Group 4).

#### Primary objective: Patterns of the exposure-response relationship of arsenic-related CVD

Person-years was computed from the date of enrollment to the date of death from any cause or December 31, 2018, whichever came first. In the Model 1, we modeled the association between cumulative arsenic exposure/arsenic concentration and risk of CVD mortality, and Cox proportional hazard models were used to calculate hazard ratio (HR) and 95% confidence intervals (CI) for each factor, adjusting for sex and baseline age groups (5-years), radon, BMI, smoking information (never, former, current), educational level (Never, Primary school and Middle school or above). The lowest exposure (Group 1) subgroup was selected as the reference group during statistical analysis. In addition, to explore patterns of the exposure-response relationship of arsenic-related CVD, we also compared the risks of a higher concentration at shorter duration with a lower concentration at longer duration within a fixed cumulative exposure. Finally, we modeled the modification effects of TSE, AA and AFE on arsenic-related CVD mortality. The median of 25.5 years for TSE and 50 years for AA were selected as the cut-off points to ensure sufficient sample size for statistical analysis, and the first exposure of 18 years old as the cut-off value to define the initial exposure in childhood (<18 years) or adulthood (≥18 years). And the subgroups (age at first exposure <18 years, attained age <50 years and time since last exposure <25.5 years) were selected as the reference group.

#### Secondary objective: Synergy between arsenic exposure and cigarette smoking on CVD mortality

The joint effect of arsenic and cigarette smoking was estimated by the synergy index (20), which was the ratio between the observed excess risk in those with exposures to 2 risk factors and the excess risk predicted under simple sum [(RR11 – 1)/(RR10 + RR01 – 2)]. To ensure sufficient sample size for statistical analysis, all miners were equally divided into three subgroups by arsenic concentration: 2,455 in 0.1–2.9 mg/m^3^, 2,696 in 3.0–8.0 mg/m^3^ and 2,584 in 8.1–100.2 mg/m^3^. In addition, current smokers were divided into two subgroups by the median of 25 pack-years. All analysis was conducted with SAS 9.4 (SAS Institute, Cary, NC).

## Results

As shown in Table 3, a total of 7,735 tin miners contributed 142,287.7 person-years of observation during which time 3,728 deaths were identified, of which 1,046 were classified as CVD deaths. Of these, 484 (46.3% of total CVD deaths) were classified as cerebrovascular diseases deaths and 388 (37.1% of total CVD deaths) as heart diseases deaths. Almost all miners were male in Group 3 and 4, and the highest proportion of female was in Group 1.

**Table 3:** Baseline characteristics and cardiovascular disease deaths among the YTC cohort participants.

	**Arsenic concentration (mg/m** ^**3**^ **)**				**Total**
	**Group 1 (0.1–2.4)**	**Group 2 (2.5–4.4)**	**Group 3 (4.5–13.9)**	**Group 4 (14.0–103.2)**	
No. of miners	1,903	1,876	2,032	1,924	7,735
Person-years	37,354.9	39,887.8	38,833.4	26,211.6	142,287.7
**Gender**					
Male	1,674	1,688	1,949	1,918	7,229
Female	229	188	83	6	506
**Education level**					
Never	360	194	319	896	1,769
Primary school	862	887	1,066	908	3,723
Middle school or above	681	795	647	120	2,243
**Age (year)**					
Median (IQR)	49 (41–59)	43 (40–50)	47 (42–56)	62 (59–66)	51 (42–61)
**BMI**					
Median (IQR)	21.4 (19.6–23.5)	21.8 (20.2–23.6)	21.6 (19.9–23.5)	20.6 (18.9–22.8)	21.4 (19.6–23.3)
**Arsenic exposure**					
**Cumulative exposure (mg/m** ^**3**^ **-year)**					
Median (IQR)	15.8 (7.6–28.5)	73.8 (56.9–90.1)	148.2 (109.9–205.5)	799.8 (586.1–1,136.9)	99.5 (45.42–361.25)
**Attained age (year)**					
Median (IQR)	69 (62–78)	45 (40–65)	46 (40–55)	48 (37–55)	50.12 (41–61.38)
**Age at first exposure (year)**					
Median (IQR)	19 (16–22)	20 (17–24)	20 (17–24)	15 (12–19)	19 (16–22)
**Time since last exposure (year)**					
Median (IQR)	24.4 (19.9–26.4)	24.4 (21.0–26.5)	26.3 (21.2–28.5)	30.8 (22.2–41.3)	25.47 (21.07–29.08)
**Smoking information**					
Never smoker	436	383	283	120	1,222
Former smoker	126	88	112	228	554
Current smoker	1,341	1,405	1,627	1,576	5,949
< 25 pack-years	776	782	885	860	3,303
≥25 pack-years	565	623	742	716	2,646
**Cause of death (ICD-10)**					
Cardiovascular diseases (I05–I69)	190	143	270	443	1,046
Cerebrovascular diseases (I60–I69)	89	74	107	214	484
Heart diseases (I05-I09, I20–I28, and I30–I52)	68	50	90	180	388
Other causes	33	19	73	49	174

IQR, interquartile range; ICD, international Classification of diseases.

All miners were equally classified as four subgroups by arsenic concentration: 1,903 participants in < 2.5 mg/m^3^ (Group 1), 1,876 in 2.5–4.4 mg/m^3^ (Group 2), 2,032 in 4.5–13.9 mg/m^3^ (Group 3), 1,924 in ≥14.0 mg/m^3^ (Group 4).

### Primary objective: Patterns of the exposure-response relationship of arsenic-related cardiovascular disease

We found an increased risk of CVD mortality with increasing levels of either cumulative arsenic exposure or concentration. The same trends were observed in mortality from cerebrovsacular and heart diseases.

For a fixed level of cumulative arsenic exposure, participants exposed to higher concentrations of arsenic over shorter duration had a higher risk of CVD mortality than those exposed to lower concentration over longer duration in participants exposed to 45.5–99.5 and 99.5–361.0 mg/m^3^. For heart diseases, the HR and 95% CI were 1.59 (95%CI: 1.01–2.49, *P* = 0.04) in participants exposed to 99.5–160.9 mg/m^3^; However, the confidence intervals are broadly over lapping and the data are not significantly different in other exposed subgroups, thus the diseases (CVD, cerebrovascular and heart diseases) mortality risks are similarly consistent for participants exposed to higher concentrations over shorter duration as compared with lower concentrations over longer duration.

In addition, for a fixed level of arsenic concentration, any significant associations were not shown between cumulative arsenic exposure and the risk of deaths from any CVD, cerebrovascular or heart diseases (As shown in Table 4).

**Table 4:** Patterns of the exposure-response relationship of arsenic-related cardiovascular disease mortality.

**The relationship between cumulative arsenic exposure and cardiovascular disease mortality**							
	**Hazard ratio (95% CI) per 50 mg/m^3^-year increase**	***P*-value**	**Hazard ratio (95% CI) by range of cumulative arsenic exposure (mg/m** ^**3**^ **-year)**				*P* for trend^*^
			**0.1–45.4**	**45.5–99.4**	**99.5–360.9**	**361.0–2,893.9**	
**Cardiovascular diseases**							
No of deaths	1,046		183	184	240	439	
Model 1	1.16 (1.11, 1.22)	< 0.01	1	1.25 (1.01, 1.55)	1.44 (1.19, 1.76)	2.01 (1.62, 2.47)	< 0.01
**Cerebrovascular diseases**							
No of deaths	484		88	73	115	208	
Model 1	1.17 (1.09, 1.25)	< 0.01	1	1.06 (0.77, 1.46)	1.43 (1.08, 1.90)	1.90 (1.40, 2.58)	< 0.01
**Heart diseases**							
No of deaths	388		65	49	85	189	
Model 1	1.25 (1.16, 1.35)	< 0.01	1	1.00 (0.68, 1.47)	1.47 (1.06, 2.05)	2.80 (2.00, 3.92)	< 0.01
**The relationship between arsenic concentration and cardiovascular disease mortality**							
	**Hazard ratio (95% CI) per 2.5 mg/m**^3^ **increase**		**Hazard ratio (95% CI) by range of arsenic concentration (mg/m** ^3^ **)**				***P*** for trend^*^
			**0.1–2.4**	**2.5–4.4**	**4.5–13.9**	**14.0–103.2**	
**Cardiovascular diseases**							
No of deaths	1,046		190	143	270	443	
Model 1	1.17 (1.12, 1.22)	< 0.01	1	0.95 (0.76, 1.19)	1.52 (1.26, 1.84)	1.84 (1.53, 2.21)	< 0.01
**Cerebrovascular diseases**							
No of deaths	484		89	74	107	214	
Model 1	1.17 (1.10, 1.24)	< 0.01	1	1.08 (0.78, 1.49)	1.30 (0.98, 1.72)	1.84 (1.41, 2.40)	< 0.01
**Heart diseases**							
No of deaths	388		68	50	90	180	
Model 1	1.20 (1.12, 1.29)	< 0.01	1	1.05 (0.72, 1.53)	1.46 (1.06, 2.01)	1.90 (1.41, 2.55)	< 0.01
**For equal cumulative exposure, higher concentrations over shorter duration vs. lower concentrations over longer duration**								
	**Hazard ratio (95% CI) and** ***P*****-value by range of cumulative exposure (mg/m**^3^**-year)**							
	**0.1–45.4**	***P*** **-value**	**45.5–99.4**	***P*** **-value**	**99.5–360.9**	***P*** **-value**	**361.0–2,893.9**	***P*** **-value**
**Cardiovascular diseases**								
No of deaths	183		184		240		439	
Model 1	0.95 (0.68, 1.35)	0.79	1.31 (0.98, 1.76)	0.07	1.33 (1.02, 1.73)	0.03	0.96 (0.78, 1.17)	0.67
**Cerebrovascular diseases**								
No of deaths	88		73		115		208	
Model 1	0.79 (0.48, 1.29)	0.34	0.94 (0.59, 1.50)	0.80	1.11 (0.76, 1.61)	0.60	0.96 (0.72, 1.27)	0.75
**Heart diseases**								
No of deaths	68		50		90		180	
Model 1	1.23 (0.68, 2.21)	0.50	0.96 (0.55, 1.69)	0.89	1.59 (1.01, 2.49)	0.04	0.93 (0.69, 1.27)	0.65
**For equal arsenic concentration, higher cumulative exposure vs. lower cumulative exposure**								
	**Hazard ratio (95% CI) and** ***P*****-value by range of arsenic concentration (mg/m**^3^**)**							
	**0.1–2.4**	***P*** **-value**	**2.5–4.4**	***P*** **-value**	**4.5–13.9**	***P*** **-value**	**14.0–103.2**	***P*** **-value**
**Cardiovascular diseases**								
No of deaths	190		143		270		443	
Model 1	0.88 (0.65, 1.21)	0.43	1.03 (0.73, 1.47)	0.85	0.98 (0.75, 1.29)	0.89	1.07 (0.88, 1.31)	0.51
**Cerebrovascular diseases**								
No of deaths	89		74		107		214	
Model 1	0.72 (0.46, 1.14)	0.16	0.87 (0.54, 1.42)	0.58	1.19 (0.77, 1.84)	0.44	1.28 (0.96, 1.71)	0.10
**Heart diseases**								
No of deaths	68		50		90		180	
Model 1	1.09 (0.65, 1.84)	0.73	1.50 (0.81, 2.76)	0.19	1.30 (0.80, 2.11)	0.30	0.98 (0.71, 1.34)	0.88

* Estimated with arsenic exposure variable as continuous variable in model.

Model 1: Adjusted for sex and baseline age groups (5-years), radon, BMI, smoking information (never, former, current), educational level (Never, Primary school and Middle school or above). For equal cumulative exposure, those with lower concentrations over longer duration were selected as the reference group. For equal arsenic concentration, those with lower cumulative exposure were viewed as reference groups.

Further, we evaluated time factors (such as AFE, AA, and TSE) as modifiers of the arsenic exposure–response relationship. Within a fixed arsenic concentration, participants with age at first exposure lower than 18 years had a significantly higher risk of mortality from CVD, cerebrovascular and heart diseases than those with 18 years or above. Similarly, the significant variations were seen in the effect modification of time since last exposure, and those with lower than 25.5 years had a higher mortality risk than those with 25.5 years or above. However, for attained age, although the HR > 1 in most exposed subgroups, there was no statistically significant variations when compared participants lower than 50 years and those older than 50 years or above. These results were shown in Table 5.

**Table 5:** Modification effects of the relationship between arsenic concentration and cardiovascular disease mortality.

	**Hazard ratio (95% CI) and** ***P*****-value by range of arsenic concentration (mg/m**^**3**^**)**							
	**0.1–2.4**	***P*-value**	**2.5–4.4**	***P*-value**	**4.5–13.9**	***P*-value**	**14.0–103.2**	***P*-value**
**Age at first exposure (**<**18 vs**. **≥18 years)**								
**Cardiovascular diseases**								
Model 1	0.67 (0.49, 0.91)	< 0.01	0.72 (0.50, 1.04)	0.08	0.53 (0.42, 0.68)	< 0.01	0.87 (0.69, 1.08)	0.21
**Cerebrovascular diseases**								
Model 1	0.51 (0.32, 0.80)	< 0.01	0.99 (0.58, 1.70)	0.96	0.60 (0.41, 0.89)	0.01	0.94 (0.69, 1.29)	0.71
**Heart diseases**								
Model 1	0.81 (0.49, 1.35)	0.42	0.59 (0.31, 1.10)	0.10	0.33 (0.22, 0.50)	< 0.01	0.54 (0.37, 0.79)	< 0.01
**Attained age (**<**50 vs**. **≥50 years)**								
**Cardiovascular diseases**								
Model 1	1.78 (0.91, 3.49)	0.09	1.56 (0.97, 2.05)	0.18	0.98 (0.71, 1.35)	0.90	0.96 (0.79, 1.16)	0.66
**Cerebrovascular diseases**								
Model 1	1.68 (0.68, 4.15)	0.26	1.37 (0.73, 2.55)	0.32	1.21 (0.73, 2.02)	0.46	1.06 (0.81, 1.41)	0.66
**Heart diseases**								
Model 1	2.27 (0.63, 8.12)	0.21	2.38 (1.21, 4.66)	0.01	1.26 (0.73, 2.16)	0.41	0.96 (0.70, 1.30)	0.78
**Time since last exposure (**<**25.5 vs**. **≥25.5 years)**								
**Cardiovascular diseases**								
Model 1	0.12 (0.08, 0.18)	< 0.01	0.11 (0.07, 0.18)	< 0.01	0.14 (0.11, 0.18)	< 0.01	0.26 (0.21, 0.33)	< 0.01
**Cerebrovascular diseases**								
Model 1	0.11 (0.06, 0.18)	< 0.01	0.11 (0.06, 0.23)	< 0.01	0.16 (0.10, 0.24)	< 0.01	0.20 (0.15, 0.28)	< 0.01
**Heart diseases**								
Model 1	0.14 (0.07, 0.26)	< 0.01	0.08 (0.03, 0.20)	< 0.01	0.12 (0.07, 0.20)	< 0.01	0.28 (0.19, 0.41)	< 0.01

Model 1: Adjusted for sex and baseline age (years), BMI, smoking information (never, former, current), educational level (Never, Primary school and Middle school or above). The subgroups of age at first exposure < 18 years, attained age < 50 years and time since last exposure < 25.5 years were selected as reference groups.

### Secondary objective: Synergy between arsenic exposure and cigarette smoking on cardiovascular disease mortality

**Figure 2:**
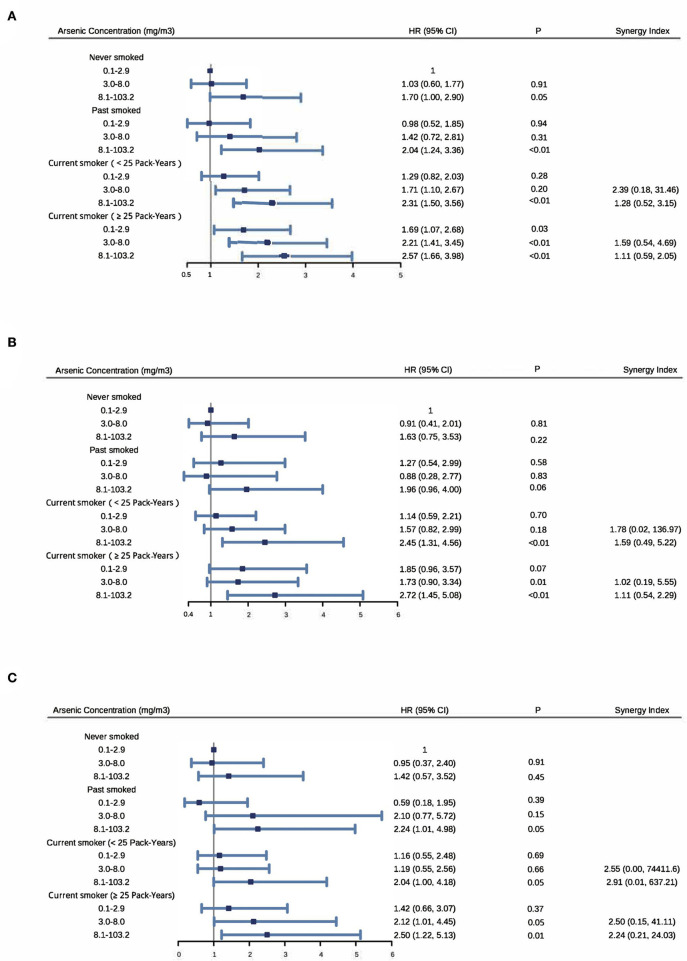
Synergy between arsenic exposure and cigarette smoking on cardiovascular disease **(A)**, cerebrovascular disease **(B)** and heart disease **(C)** mortality. Adjusting for sex and baseline age groups (5-years), radon, BMI and educational level (never, primary school and middle school or above). Participants were equally divided into three subgroups by arsenic concentration: 2,455 in 0.1–2.9 mg/m^3^, 2,696 in 3.0–8.0 mg/m^3^ and 2,584 in 8.1–100.2 mg/m^3^. Among current smokers, they were divided into two subgroups by the median of 25 pack-years.

As shown in Figure 2, among nonsmokers, those who were exposed to the highest arsenic level (8.1–103.2 mg/m^3^) had an increased risk of cardiovascular death (HR: 1.70; 95% CI: 1.00, 2.90), cerebrovascular death (HR: 1.63; 95% CI: 0.75, 3.53) or heart death (HR: 1.42; 95% CI: 0.57, 3.52), when compared with those with the lowest level (0.1–2.9 mg/m^3^). Among participants with the lowest arsenic level, those who had 25 or more pack-years of smoking had an increased risk of cardiovascular death (HR: 1.69; 95% CI: 1.07, 2.68), cerebrovascular death (HR: 1.85; 95% CI: 0.96, 3.57) or heart death (HR: 1.42; 95% CI: 0.66, 3.07), compared with nonsmokers. When compared with nonsmokers with an arsenic exposure level among 0.1–2.9 mg/m^3^, those who with an arsenic level of 8.1–103.2 mg/m^3^ and smoked for more than 25 pack-years had an increased risk of cardiovascular death (HR: 2.57; 95% CI: 1.66, 3.98), cerebrovascular death (HR: 2.72; 95% CI: 1.45, 5.08) or heart death (HR: 2.50; 95% CI: 1.22, 5.13). Among participants with the highest arsenic level, HR >1 for all three outcomes regardless of smoking status.

In addition, 15%−43% of cardiovascular death, 1%−68% of cerebrovascular death and 20%−74% of heart death were attributable to both arsenic exposure and cigarette smoking. Furthermore, all synergy indices were >1 [range: cardiovascular death (1.11–2.39), cerebrovascular death (1.02–1.78) and heart death (2.24–2.91)] indicating the existence of synergism in an additive way (Figure 2).

## Discussion

Based on this occupational cohort with a long-term follow-up time, we explored the patterns of the exposure-response relationship between airborne arsenic exposure and CVD mortality and investigated the synergy effect of cigarette smoking. Data showed that participants with <18 years at first exposure, <25.5 years since last exposure and smoking were more prone to deleterious effect of airborne arsenic exposure.

Consistent with other previous studies (21–26), we found that either cumulative arsenic exposure or arsenic concentration was associated with increased risk of deaths from CVD, cerebrovascular or heart diseases. However, this is the first study that generated further evidence to affirm the patterns of the exposure-response relationship of arsenic-related CVD. Results showed miners exposed to higher concentrations over shorter duration had an increased mortality risk from CVD than those exposed to lower concentrations over longer duration, but they were not significantly different. Even so, they still provided noteworthy clues for policy makers that more attention should be paid to those with higher concentration over shorter duration (27). This pattern may be closely related to the mechanism of toxicity of arsenic exposure. The arsenic toxicology on human largely depended on the capacity of cells to methylate and detoxify. That methylated forms had a greater affinity to bind protein thiol sites than nonmethylated arsenicals, which leading to disruption of cell function (28–30). Studies of arsenic in drinking water showed the ability of arsenic excretion in healthy individuals increased and the methylation efficiency of ingested arsenic decreased, when the arsenic concentration was reduced to lower levels (31, 32). However, we did not observed a statistically difference between two different patterns for the risk of deaths from any CVD, cerebrovascular or heart diseases, which might be explained by the fact that there still existed arsenic exposure in reference group due to the inclusion criteria of a confirmed exposure history of at least 10 years.

This cohort firstly reported that the arsenic-related risk of deaths from CVD from initial exposure in childhood (<18 years) was greater than when first exposure occurred at adulthood (≥18 years), which further strengthened evidence from a cohort study in Matlab, Bangladesh with 13 years follow-up and <40 CVD death cases (33). In the cohort study in Matlab, researchers reported that higher concentration of arsenic in drinking water increased the mortality risk among the young adults, but cerebro-vascular disease, cardio-vascular disease, and respiratory disease as a whole were used to analyzed. Although compelling evidence suggested that young adults after exposure to arsenic in early childhood conferred increased risk from diseases such as all death causes (34), lung cancer (35), bladder cancer (36, 37), CVD (33) and airway allergy (38), but there were no studies directly comparing arsenic-related risk from diseases in childhood and adulthood. Notably, with 27 follow-up years and 1,046 death cases from CVD, this is the first study to generate further evidence that childhood exposed to arsenic have an increased risk of CVD, cerebrovascular or heart death than adulthood exposed to arsenic. Humans were extremely susceptible to early-life hazardous exposure. For example, early-life exposed to substances such as asbestos, radiation, and diethylstilbestrol have been unequivocally linked to adult cancer in human studies (39). A possible reason is that in early childhood periods, rapid organogenesis and cell proliferation allow for mutagenic, epigenetic alterations, and metabolism, detoxification, and excretion pathways were undeveloped (40). However, more evidence from epidemiological, molecular and animal studies is still needed in this field. From a public health perspective, the intervention measure that protect childhood from arsenic exposure will be cost-effectiveness when resources are constrained.

In addition, our data suggested that arsenic-related risk of deaths from CVD, cerebrovascular or heart diseases declined with time since last arsenic exposure. For the long-term health impacts of arsenic exposure, an ecological study in Northern Chile showed that arsenic exposure had very long latency, because the risks of lung, bladder, and kidney cancers still increased at least 40 years after exposure reduction (41). In our cohort, after arsenic exposure cessation, those miners within 25.5 years during the follow-up period had a higher mortality risk than those with 25.5 years or above. That is, for individuals with arsenic exposure cessation, adverse health effects will persist at least 40 years but the effects might significantly decline with years since exposure cessation.

Our data suggested that cigarette smoking increased susceptibility to the CVD effects of arsenic, which was similar to findings from the Health Effects of Arsenic Longitudinal Study in Bangladesh (9) and the New Hampshire Skin Cancer Study (8). Evidences from previous studies showed that the joint effect of arsenic exposure and cigarette smoking on incidence and mortality from CVD was greater than the sum of their individual effects. The main reasons may be that smoking status would impact the methylation ability of arsenic. On the one hand, compared with nonsmokers, a higher ratio of urinary monomethylarsonate (MMA) to dimethylarsinate (DMA) in smokers has been consistency related to other chronic diseases like cancers (32). On the another hand, cigarette smoking could increase the requirement of folate, as is shown by the China Stroke Primary Prevention Trial that compared with never smokers, ever smokers may require a higher dosage of folic acid to achieve a greater beneficial effect on stroke (42). However, folate as a critical co-factor plays a key role in the one-carbon metabolism, a process through which arsenic is enzymatically methylated. Given cigarette smoking is likely to influence arsenic toxicity, studies of lower levels of arsenic exposure should take smoking information into consideration. In our study, only if participants are exposed to both cigarette smoking and arsenic exposure at concentrations as low as <3.0 mg/m^3^, and they would have a higher risk of CVD deaths compared with reference group.

In addition, the synergy between smoking and arsenic exposure on CVD deaths persists either in light smokers or heavy smokers. Moreover, for equal arsenic concentration, variations in CVD deaths are not statistically significant when compared light smokers with heavy smokers. More and more evidences support that smoking cessation, but not reduction, is associated with reduced CVD risk (43–46). Therefore, to reduce the burden of CVD in population, measures of sustained quitting and minimize arsenic concentration should be advocated.

A major strength of this study was that a clear-cut occupational arsenic exposure pattern made it possible to evaluate the relationship between lifetime exposure and CVD deaths. For retired miners in our cohort, occupational arsenic exposure was negligible because miners left the workplace which was the main resource of pollutant. In addition, we tracked changes in smoking status during the initial four years of follow-up, which strengthened the stability of smoking information. Lastly, this cohort over 20 years of follow-up with little loss to follow-up had relatively large sample size, which resulted in sufficient statistical power in subgroup analysis. This study also had several limitations. Inclusion criteria with 10+ years of arsenic exposure history meant that exposed miners were be served as a reference category, which would underestimate our estimates of the arsenic effect. Secondly, in our cohort, the high smoking rate (76.9%) resulted in significant passive smoking exposure, even for non-smokers. Therefore, the use of “non-smokers” with such exposure would also bias estimates toward the null. Finally, individual miners' arsenic exposure was obtained through the individual exposure time and airborne dust concentrations in workplace, but there was no validation or quality control of different collection methodologies at different times. Therefore, the uncertainty of this estimation might bias our results, and urine arsenic concentrations should be measured in the future studies.

In summary, for equal cumulative arsenic exposure, more attention should be paid to protect population with higher concentration over shorter duration from CVD mortality. And, more attention should also be given to those with <18 years at first exposure, <25.5 years since last exposure and tobacco users.

## Data availability statement

The raw data supporting the conclusions of this article will be made available by the authors, without undue reservation.

## Ethics statement

This study was approved by the institutional review boards of the National Cancer Center/National Clinical Research Center for Cancer/Cancer Hospital, Chinese Academy of Medical Sciences (201812190401002). The patients/participants provided their written informed consent to participate in this study.

## Author contributions

Y-LQ and F-HZ had full access to all the data in the study and take responsibility for the integrity of the data and the accuracy of the data analysis. Conception and design: Q-HZ and Y-LQ. Data collection: ZS, Y-GF, and Q-HZ. Analysis, interpretation, and drafting the article: ZS and X-HJ. Manuscript revision: Y-GF, Q-HZ, and Y-LQ. All authors contributed to the article and approved the submitted version.
