# Supplementary material for: Synergy of arsenic with smoking in causing cardiovascular disease mortality: A cohort study with 27 follow-up years in China
Source: Front Public Health. 2022 Dec 14;10:1012267. doi: 10.3389/fpubh.2022.1012267 (PMC9795054; doi:10.3389/fpubh.2022.1012267)
Supplement: Supplementary file 1 [file Table_1.DOCX]

**Table S1.** Total causes of death and data sources in this cohort

|  |  | **Data Sources** | | | | |
| --- | --- | --- | --- | --- | --- | --- |
| **Cause of Death** (ICD-10)* | Numbers of Miners | Local cancer registration agency | Medical record system | Death cause systems of public security bureau | Death cause systems of funeral parlor | Face-to-face interviews |
| Cardiovascular diseases (I05-I69) | 1046 | / | 943 | 50 | 6 | 47 |
| Lung cancer (C34) | 921 | 808 | 63 | 13 | 16 | 21 |
| Other cancers | 384 | 315 | 47 | 3 | 7 | 12 |
| Diseases of the Respiratory System (J00-J99) | 499 | / | 447 | 26 | 12 | 14 |
| Diseases of the Digestive System (K00-K93) | 180 | / | 155 | 13 | 7 | 5 |
| Other causes | 406 | / | 322 | 56 | 18 | 10 |
| Not available | 156 | / | / | / | / | / |

*: ICD: international Classification of diseases.
